# Supplementary material for: Are carbon emissions trading and green financial instruments synergistic? -Comprehensive quantitative research based on content analysis
Source: PLoS One. 2024 Mar 7;19(3):e0298601. doi: 10.1371/journal.pone.0298601 (PMC10919601; doi:10.1371/journal.pone.0298601)
Supplement: S1 Data — (ZIP) [file pone.0298601.s001.zip › Data/地级市Z_F工作报告.zip!/╜¡╦╒/╠⌐╓▌╩╨/└┤╫╘╣1⁄2╓┌║┼í╛┬φ┐╦╩2╛▌═°í┐_2008.docx]

各位代表：

现在，我代表市人民政府向大会作工作报告，请予审议，并请市政协委员和其他列席人员提出意见。

一、过去五年工作回顾

2003年至2007年是泰州全面建设小康社会的重要时期。我们在中共泰州市委领导下，坚持以邓小平理论和“三个代表”重要思想为指导，认真贯彻落实科学发展观和构建和谐社会的重大战略思想，紧紧依靠全市人民，励精图治，奋发进取，全市改革开放和现代化建设取得了重大成就，圆满完成了市二届人大会议确定的目标任务。

过去五年，是综合实力大幅提升的五年。五年来，面对整个宏观发展环境趋紧的形势，积极采取有效措施，促进经济又好又快发展，提前和全面完成“五年总量翻番”任务。预计2007年地区生产总值1200亿元，是2002年的2.4倍，年均可比增长14.9%；财政收入210亿元，其中地方一般预算收入85亿元，分别是2002年的3.95倍和3.88倍；二、三产业增加值占GDP的比重达91.5%，比2002年提高5.4个百分点。扎实推进农业结构调整，农业综合生产能力进一步提高。以八大产业为主导的农业区域特色经济初步形成，粮食品种优质化率、特种养殖规模化水平位居全省前列，高效农业面积扩大到120万亩；农业产业化、市场化建设成效明显，累计新增农副产品加工企业800家、各类合作经济组织650家、农业利用“三资”100亿元；农业科技服务、动植物疫病防控等支持保障体系进一步完善，农业保险工作启动实施，农业抗击风险的能力得到增强；市农业开发区被列为省级现代农业综合开发示范区，基础设施建设和产业项目招商取得积极进展。大力推进工业强市“第一方略”，工业主体地位进一步凸现。重点发展医药、机电、船舶、化工四大优势产业和不锈钢制品等十大特色产业集群，2007年四大优势产业实现销售突破千亿元，达1250亿元；先后培育工业企业“30强”、“50强”,扬子江药业集团年销售超百亿元、陵光集团等4家企业超50亿元；坚持每年抓好一批投入过亿元重大技改项目的滚动实施，工业技改投入持续实现两年翻一番，累计达1188亿元；新认定国家级技术中心3家、省级21家。全市规模以上工业实现销售突破2000亿元，预计达2120亿元，是2002年的4.3倍；增加值年均增长22.4%，连续4年位居全省前列。建筑业经济总量保持全省领先水平，泰兴市、姜堰市被省政府命名为建筑强县（市），江苏一建等4家企业进入全省综合实力20强。突出抓好服务业重点项目建设、重点企业和集聚区发展，服务业发展速度进一步加快。区域物流、商贸餐饮、金融保险、社区服务、休闲旅游等行业呈现出较快发展势头，金鹰国际、中央商场等知名商贸企业相继入驻，泰州医药高新技术产业园区综合物流园区、泰州城北物流园区、江苏三江现代物流中心被列为全省重点物流基地，溱湖风景区被评为国家AAAA级风景区和国家湿地公园，凤凰河风景区被评为国家级水利风景区。2007年社会消费品零售总额318亿元，比2002年翻了一番；年末各项贷款余额668亿元，比2002年末净增378亿元。在2006年全国综合实力百强城市评比中，我市列第47位，五年前移91位。

过去五年，是改革开放取得突破的五年。五年来，围绕进一步增强经济发展的动力和活力，坚持不懈地推进改革、扩大开放。在全省率先全面完成工贸企业改革改制任务，机制和体制性增长的潜力得到充分释放。鼓励、支持和引导民众创业，民营经济蓬勃发展。五年净增私营企业1.4万家，净增个体工商户2.4万户；2007年末私营个体经济注册资本达636.6亿元，是2002年末的6.1倍。经营性服务类事业单位改制任务基本完成，公益类事业单位改革试点稳步推开。以取消农业税为重点的农村综合改革逐步深化，财政管理体制和投融资体制改革取得积极进展。开放型经济实现跨越式发展。预计五年累计实际利用外资28.3亿美元，增速一直位居全省前列，其中2007年实际利用外资8.5亿美元，是2002年的4.7倍；累计完成自营出口79.3亿美元，年均增长46.1%，其中2007年自营出口达28亿美元，是2002年的6.7倍。实施“走出去”战略，境外工程承包和劳务合作总量跃至全省第4位，姜堰双登、兴化兴达、靖江东方重工在境外成功上市。沿江开发势头强劲，跨江联动率先突破。泰州电厂等一批重大产业项目成功落户并竣工投产，沿江高等级公路等一批重点基础设施项目如期建成。市经济开发区和重点工业园区已成为全市招商引资的主阵地，江阴—靖江工业园、海陵工业园、高港高新技术产业园被批准为省级经济开发区，泰州医药高新技术产业园区起步良好。泰州边检站开检运行，口岸远程监控系统建成,“大通关”建设取得新进展，边检、国检、海关、海事等口岸查验机构为开放型经济发展作出了积极贡献。加强内、外交流合作，先后举办三届科技经贸洽谈会，成功承办亚洲博鳌论坛国际医药产业大会和长三角城市经济协调会第七次会议，与8个外国城市缔结为友好城市，外事、侨务和港澳台事务工作取得新成绩，泰州知名度和影响力得到进一步提升。

过去五年，是城乡面貌变化巨大的五年。五年来，始终坚持统筹发展理念，扎实推进城乡建设，着力优化发展环境。调整、理顺市区城建体制，坚持新、老城区建设并举，海陵、高港发展并进，完善功能、提升品位并重，突出抓好三年实施纲要推进和年度十大工程建设，中心城市人居环境显著改善，集聚和辐射能力明显增强。五年城建累计投入300多亿元，建成区面积由2002年的40平方公里扩大到56平方公里。泰州火车站、市文化中心、高教园区等标志性工程如期建成或开工建设，青年路等骨干道路改造及延伸、坡子街等重点商业街区建设、后街背巷和城北地区改造顺利实施，城市防洪、管道燃气及污水与垃圾处理等基础设施日趋完善。凤城河风景区一期、泰山公园改扩建、乔园改扩建一期、城隍庙修复、学政试院修缮、高港生态公园一期、雕花楼以及一批城市小游园等景区、景点建设工程相继完工，城市公共绿地面积累计增加383万平方米。组织编制城市控制性详细规划和专项规划，推进城乡规划全覆盖，相对集中城管领域行政处罚权。开展“五城同创”和新“五城同创”，成功创建成国家卫生城市、国家环保模范城市、全国双拥模范城、江苏省园林城市和中国优秀旅游城市。各辖市普遍加大城区建设力度，进一步拉开框架、完善功能、提升形象，城区面貌发生很大变化。全市城市化率由2002年的40.9％上升到48％。新农村建设取得阶段性成果。组织实施全面小康“十、百、千”工程，镇村布局规划编制完成，农村环境综合整治成效明显，200个行政村创建成市级全面小康先行村，姜堰市沈高镇河横村成为全省唯一的“全国部省共建新农村建设示范点”。城乡基础设施进一步完善。宁启铁路泰州段建成通车，姜八公路扩建等一批干线公路基本建成，泰州长江大桥、兴泰公路改扩建等工程开工建设，全市大交通格局初步形成。市区长江水达镇工程实施到位，惠及百万群众的区域供水工程开工建设，农村饮用水安全工程启动实施，江堤达标建设、里下河地区灾后重建等重点工程相继建成，农村公路累计新（改、扩）建5000公里，村村通公路目标如期实现。电力、通信等基础设施建设取得重大进展，公用电网建设累计投入50.8亿元，区域性无线电监测中心建成。预计五年累计完成全社会固定资产投资2153亿元，年均增长30.9%，其中2007年完成680亿元，是2002年的3.8倍。积极打造绿色泰州、生态泰州，开创环境信访听证全国先河，强化岸线、土地资源节约利用和环境保护，提前完成省交关闭100家小化工企业任务，全市环境质量综合指数位居全省前列，所辖市全部创建成国家级生态示范区。

过去五年，是社会建设全面推进的五年。五年来，在加快经济发展的同时，切实加强社会建设，努力促进社会进步。大力推进“科教兴市”战略，积极打造创新型城市。五年累计实施省级以上火炬计划192项、星火计划117项，其中国家级分别达112项和47项；开发省级以上高新技术产品404项，其中国家级重点新产品85项；新增省级以上高新技术企业182家，其中国家级高新技术企业42家；获得省科技进步奖25项、国家科技进步奖5项。我市被评为“全国科技进步先进市”，被列为“国家科技兴贸生物医药出口创新基地”。人才队伍建设不断加强，共引进高层次人才2890人。贯彻教育优先发展方针，深入推进素质教育。扎实抓好农村义务教育管理体制改革、中小学布局调整、合格学校建设和“两免一补”政策落实，义务教育阶段入学率、巩固率分别达100%和99.8%；大力普及高中阶段教育，高考万人本科进线率位居全省前列；加快高等教育发展，建成南理工泰州科技学院、南师大泰州学院，结束了泰州无本科教育的历史，江苏畜牧兽医职业技术学院、泰州职业技术学院、泰州师范高等专科学校先后通过教育部“人才培养工作水平评估”并获优秀等次，5所高校在校生已达3.5万人；积极发展职业教育，创成省级以上重点职业（技工）学校17所。重视和加强文化建设，大型现代淮剧《祥林嫂》的主要扮演者获得第21届中国戏剧梅花奖，淮剧《信访局长》获得第五届江苏省淮剧节优秀剧目一等奖，全市新增国家级文物保护单位3处、省级8处,“溱潼会船”等5个项目入选首批省级非物质文化遗产名录，辖市（区）文化基础设施建设都取得重要进展。健全公共卫生体系，改善基本医疗服务，抗击非典和防治禽流感取得胜利。市传染病医院、市疾控中心、市妇幼保健院、高港区人民医院（迁建）和普济医院相继建成，市人民医院创建成三级乙等医院，市中医院创建成三级甲等中医院。大力推进有线广播电视进村入户，综合入户率达80%。全民健身和竞技体育取得新成绩，实施“万村体育健身工程”，行政村基本建成村级体育健身场地；成功承办十运会男子篮球比赛等多项赛事，两名泰州籍运动员获得世界冠军。出台计划生育社会保障办法，人口和计划生育工作跻身全省先进地区行列。平安泰州建设扎实推进，突发事件应急处置机制进一步健全和完善。我市被省委、省政府确定为“社会治安安全市”，四市两区先后被确定为“社会治安安全县（市、区）”，人民群众对社会治安的满意率达98%。强化安全生产，各类安全生产事故起数和死亡人数连续5年“双下降”。加强国防教育，兵员征集、民兵预备役、双拥等工作取得新成绩。建成全省一流的市级人防指挥所，我市被表彰为“全国人防建设先进单位”。扎实推进群众性精神文明创建，组织实施《全民科学素质行动计划纲要》，着力打造“百姓议事园”、“百姓大学堂”等“百姓”系列市民教育品牌，积极开展“爱心奶奶”、“关爱留守儿童”等“爱心”系列文明关爱活动，市民素质和城市文明程度明显提升。新闻、统计、史志档案、社会科学、气象、防震和民族宗教、妇女儿童、残疾人、红十字等工作得到加强，质监、药监、工商管理在服务地方经济社会发展中发挥了积极作用。

过去五年，是改善民生成效显著的五年。五年来，始终坚持“发展经济，造福百姓”的理念，自觉实践为民宗旨，竭尽所能改善民生。出台就业促进政策，组织实施创业带动就业行动、充分就业社区创建活动和百万农民培训工程，累计净增城镇就业人员15.9万人，下岗失业人员再就业11.3万人。加强社会保障体系建设，养老、医疗、失业保险覆盖面超过省定小康标准，新型农村合作医疗参合率达94.8％，城乡低保实现应保尽保，农村基本养老保险在百村试点。我市被国务院授予“全国再就业先进单位”，被列为全国城乡统筹就业、城镇居民基本医疗保险和“金保工程”试点市。高度重视经济薄弱地区发展和困难群众生活安排。组织实施经济薄弱乡镇“221工程”，5年帮扶任务基本完成；开展“南户北村”帮扶和机关干部结对帮扶活动，累计救助市区特困家庭5680户，扶持农村贫困户4.8万户；政府差额购买就业岗位，切实帮助“4045”人员、零就业家庭以及特殊困难群体就业再就业，对低保户和特困家庭发放物价补贴；建立大病医疗救助基金，成立泰州市惠民医院，开展“快乐从心开始”、“为了母亲的微笑”和“关爱女孩行动”等医疗救助活动；在全省率先实现义务教育免收学杂费城乡全覆盖，着力构建覆盖各类教育的扶贫助困体系，全市无一名学生因贫失学；启动廉租住房保障工作，推行“产权共有、租售并举”办法，进一步扩大经济适用房和住房公积金制度的惠及面；建立被征地农民基本生活保障制度，强化农民工维权和困难群体法律援助工作。坚持每年为市区人民办十件实事，与群众切身利益紧密相关的一些问题得到较好解决；先后组织推进农村“5+1”实事和新“5+1”实事工程，农民生产生活条件明显改善；启动实施改善民生十大工程，努力让发展成果更多地惠及广大群众。2007年城镇居民人均可支配收入达14980元，农民人均纯收入达6450元，年均分别增长14％和11％。城镇和农村居民人均住房面积分别由2002年的23平方米、40.6平方米增加到35平方米和42平方米，城乡居民存款余额从336亿元增加到640亿元，恩格尔系数由42%下降到40%，人民生活从温饱型加快向全面小康型迈进。

过去五年，是自身建设不断加强的五年。五年来，紧紧围绕打造服务政府、高效政府、法治政府、廉洁政府，进一步转变职能、深化改革、加强管理，扎实抓好自身建设。坚持为民勤政，强化宗旨教育和保持共产党员先进性教育，公务员队伍整体素质不断提高，涌现出以全国重大典型张云泉为代表的一批先进模范；持续开展以“为发展服务，让群众得益”为主题的基层站所作风建设活动和万人评机关活动，部门服务水平明显提高，发展环境不断优化；开展领导干部下访活动，强化信访工作责任制，及时、妥善处置各类信访案件和矛盾纠纷，办好市长公开电话、市长信箱和行风热线，群众的一些实际问题和困难得到较好解决，信访工作多次受到省委、省政府表彰。坚持科学施政，出台《关于推进重大决策科学化民主化的实施意见》，涉及民生的决策事项广泛听取群众意见，邀请市民代表列席市政府常务会议；组织实施“411工程”和“六个倍增计划”，推进政府工作目标管理；强化经济发展宏观指导，出台应对调控政策指导意见，有效破解“四紧”难题。坚持依法行政，自觉接受市人大、市政协的监督，重视发挥民主党派、工商联和人民团体的作用；健全联系市人大代表和市政协委员工作制度，认真办理人大代表建议和政协委员提案，综合满意率达99.3％；出台《关于对行政权力运行实施有效制约和监督的意见》，在全省率先推行政府信息公开和行政权力公开透明运行试点，保障群众的知情权、监督权；贯彻实施《行政许可法》，深化行政审批制度改革，市行政服务中心建成运行。坚持廉洁从政，出台《建立健全惩治和预防腐败体系的实施办法》，开展反腐倡廉主题教育系列活动，健全和完善工程建设招投标、土地使用权出让、政府采购等制度，推进医药购销、教育乱收费、涉农涉企收费等纠风和专项治理工作，严肃查处各类违法违纪案件，切实加强审计监督，政风、行风建设取得新成效。加强机关事务管理，保障和服务能力进一步增强。

各位代表，过去五年我们走过了不平凡的历程，取得了令人瞩目的成就。这是中共泰州市委正确领导的结果，是市人大、市政协关心支持的结果，是全市广大干部群众共同努力的结果，也得益于第一届政府艰苦创业打下的良好基础。在此，我代表本届人民政府，向辛勤工作在全市各行各业的广大干部群众，向给予政府工作热情支持和有力监督的人大代表、政协委员，向各民主党派、工商联、无党派爱国人士、各人民团体和离、退休老同志，向各驻泰机构及驻泰部队指战员、武警官兵和公安干警，向所有关心支持泰州现代化建设的海内外朋友，致以崇高的敬意并表示衷心的感谢！

过去的五年，全市经济建设和社会发展虽然取得了长足进步，但仍存在一些不容忽视的问题和不足，主要是：经济结构性矛盾依然比较突出，节能减排和环境保护的任务仍很艰巨，农业持续增效和农民持续增收的基础不够稳固，经济国际化程度和对外开放水平仍然不够高，中心城市集聚和辐射带动作用有待进一步增强，社会建设和文化建设等方面的基础还比较薄弱，涉及群众切身利益的一些突出问题尚需有效解决，政府行政能力和服务水平也有待进一步提升等。这些问题和不足，必须采取更为有效措施，在今后的工作中切实加以解决。

二、今后五年的奋斗目标和主要任务

各位代表，今后五年是泰州全面建成小康社会的关键时期，也是泰州加快发展的重要战略机遇期。我们要高举中国特色社会主义伟大旗帜，以邓小平理论、“三个代表”重要思想为指导，全面贯彻党的十七大精神和胡锦涛总书记对泰州工作的重要指示，紧紧围绕市第三次党代会和市委三届四次全会的决策部署，深入贯彻落实科学发展观，继续解放思想，深化改革开放，推进科学发展，着力改善民生，努力建设“经济发达、文化繁荣，生活富裕、生态良好，政治清明、社会和谐”的新泰州。

今后五年的奋斗目标是：围绕“全面达小康、建设新泰州”的总任务，在2010年全市总体上建成全面小康社会的基础上，按照“建设标准更高、内涵更加丰富”的要求，努力建成不含水分、群众得到实惠、老百姓认可的更高水平的小康社会。

今后五年的发展定位为：

——努力成为苏中率先发展重要板块。综合实力进一步提升，2012年地区生产总值比2007年翻一番，总量超2500亿元，人均GDP突破5万元；财政收入450亿元以上，其中地方一般预算收入力争180亿元；五年全社会固定资产投资累计达5500亿元，工业技改投入累计达3700亿元，实际利用外资累计超60亿美元。发展速度进一步加快，主要经济指标增幅在全省乃至长三角城市中处于领先地位。发展特色进一步凸现，成为苏中先进制造业基地、江苏沿江开发重点发展区、锡常泰城市群重要一极。经过五年努力，在全国综合实力百强城市中的排名进一步前移。

——努力成为全省科学发展先行地区。经济发展方式转变取得重大进展，产业结构进一步优化，2012年二、三产业增加值占GDP的比重达95%；区域功能分区进一步明晰，着力构建产业园区、城镇片区、现代农业区、生态保护区等区域板块，成为全省区域功能特色化发展的示范区；自主创新能力进一步提升，加快由泰州制造向泰州创造转变，率先成为全省创新型城市；资源集约利用进一步强化，循环经济规模位居全省先进城市行列。城乡统筹、协调发展取得重大进展，以工促农、以城带乡的长效机制逐步建立，城乡经济社会一体化发展格局初步形成，力求走出一条具有泰州特色的城乡发展新路子。生态文明建设取得重大进展，污染治理、环境保护和安全得到切实加强，区域环境质量综合指数保持全省领先地位，总体上在全省率先进入环境优化的发展新阶段。

——努力成为全国和谐发展知名城市。改善民生十大工程取得新成效，社会就业更加充分，社会保障基本实现城乡居民全覆盖，绝对贫困现象得到消除，民生“五有”目标全面实现，成功打造全国“爱心城市”。文明诚信泰州建设取得新成效，市民文明素质明显提高，诚实守信社会风气更加浓厚，干群关系、人际关系更为融洽，创成全国创建文明城市工作先进市，成为全国信用良好区域、文明和谐地区。民主法治泰州和平安泰州建设取得新成效，人民群众在经济、政治、文化等方面的权益和社会公平正义得到切实保障，成为全国最安全地区之一。2012年人民幸福指数大幅提高，城市和谐发展指数进入全国先进城市行列。

落实上述发展要求，今后五年的主要任务是：

1、加快转变发展方式，努力实现经济又好又快发展。坚持走具有泰州特色的新型工业化道路，重点发展“四高”产业，着力构筑以高效农业为基础、高端制造业和高成长性服务业为主体、高新技术产业为先导的先进产业体系。五年内，农业“5218工程”成效明显,“五横两纵”高效农业产业带、以市农业开发区为核心的市区高效农业板块和农业“八大产业链”初步形成，2012年全市高效农业面积占比达35％以上、畜禽规模养殖占比达75%以上；推进一乡一品、一市（区）一业，逐步使每个辖市能形成至少一个年产值过10亿元的农业优势特色产业。工业主导产业进一步向产业链高端攀升，规模化水平、产业集聚度明显提高，2012年医药、机电、船舶、化工四大优势产业实现销售达3300亿元，不锈钢制品等十大特色产业集群实现销售过1500亿元，形成年销售超百亿元或利税过10亿元企业10家。“833工程”取得重大进展，形成大物流、大市场、大商贸、大旅游的服务业发展新格局，基本建成区域性物流中心、商贸中心和长三角新兴休闲度假旅游基地、全国医药服务外包基地，2012年服务业增加值占GDP的比重达39％以上。推进新兴产业规模化，以新医药、新材料、新能源、新电子为重点的高新技术产业加快发展，2012年高新技术产业产值占规模以上工业的比重达30％以上。坚持走集中、集约、集聚式发展之路，积极推进“项目飞地布局、拆迁人口飞地安置、开发园区飞地联动”，“三沿”发展战略深入实施，跨江联动开发取得新成效，区域经济向差别化、特色化方向发展。坚持走以应用研发为特点的自主创新之路，大力开展产学研合作，科技人才队伍建设、科技成果转化、科技公共服务平台建设等关键问题取得重大突破，2012年全社会研发投入占GDP的比重力争达2%。坚持走质量兴业之路，进一步提升质量管理水平，争创全国质量兴市先进市。

2、深入推进改革开放，进一步增强发展动力和活力。始终不渝地把改革开放作为推动跨越发展的强大引擎，加大重点领域和关键环节的改革攻坚力度，全面提升经济国际化水平。五年内，农村综合配套改革继续深化，农民土地经营权流转、农业社会化服务体系和农村专业经济合作组织建设取得新进展；现代市场体系建立健全，公平开放、竞争有序的市场环境初步形成；行政管理体制和社会管理体制改革加快推进，公共服务型政府建设取得新成效。民众自主创业活力明显增强，民营经济发展速度进一步加快，注册资本年均增长20%以上。经济国际化程度明显提高，对外开放广度和深度得到拓展，招引产业关联度大、技术层次高、市场覆盖面广、国家鼓励和支持的基地型、龙头型大项目取得重大突破，注册协议外资超亿美元项目10个以上；市经济开发区等8个省级园区集聚、创新和产出功能明显增强，泰州医药高新技术产业园区建设和招商取得重大进展；外贸增长方式实现转变和升级，自主品牌产品和机电产品、高新技术产品出口进一步扩大，2012年自营出口50亿美元以上；实施“走出去”战略成效明显，力争培育一批主业突出、竞争力强的跨国公司。

3、致力统筹城乡建设，不断改善区域发展环境。围绕“三年形成大城市框架，五年展现大城市形象”的目标，有序拓展城市发展空间，完善优化重大功能分区，促进组团城区协调发展，进一步做大、做强、做优、做美中心城市。五年内，中心城市建设累计投入800亿元，重点实施周山河街区、城北街区、火车站街区和市文化中心、园博园、市体育中心等一批重大工程，继续加强城市道路、基础设施和景区景点建设。到2012年,“一城三区”组团发展格局基本形成，中心城市建成区面积扩大到90平方公里，城市载体功能和综合服务功能更加完善，“水亲、绿透、文昌、城秀”的城市风貌进一步凸现，跻身长三角最适宜人居、创业和发展的现代化城市行列。加快辖市城区建设步伐，引导重点中心镇向小城市和城市片区方向发展,“一大三中两小多片”的市域城镇体系、大中小城市和小城镇协调发展的新格局初步形成。2012年城市化率提高到57%。高起点推进村庄规划和建设，农村生产区、居住区、生态区和重大基础设施空间布局进一步优化，原生态村庄的生态特色和自然风貌得到保护、提升，农村发展环境和农民生产生活条件大为改善,“千村小康”目标实现。统筹推进重大基础设施建设，引江河大道开工建设，高速绕城、快速环城的城市道路体系基本形成；周山河整治、泰东河拓浚等重点水利工程如期实施，区域供水工程近期目标全面完成；泰州长江大桥及北接线、宁启铁路泰州段复线及电气化改造、京沪和宁通高速公路泰州段扩容、江海高速公路泰州段、兴泰公路改扩建等重点工程基本建成，泰州电厂二期、苏中机场力争开工建设。

4、更加注重社会发展，着力保障和改善民生。把民生事业放在更加突出的位置，以构建“社会建设六大体系”为重点，以实施改善民生十大工程为抓手，让人民群众实实在在地感受到生活每年都有新改善，一年更比一年好。坚持富民优先，促进就业惠民、创业富民、社保安民，持续增加城乡居民收入。2012年城镇居民人均可支配收入、农民人均纯收入分别达26000元和10000元。完善就业促进政策，强化就业社会服务，帮助困难群体就业，2012年城镇登记失业率控制在3％左右。加快城乡社会保障一体化进程，健全社会保险体系，2012年城镇养老、医疗、失业保险综合覆盖率达99％以上，农村基本养老保险覆盖率达40％以上，新型农村合作医疗参合率稳定在95％以上。进一步整合社会救助资源，完善城乡居民低保标准、五保老人供养标准自然增长机制和城镇低收入家庭住房保障制度，到2010年城乡困难群众每人每天生活费不低于1美元，保证城区低保家庭住得上廉租房、低收入家庭住得起经济适用房、新就业人员租得起房。建立普惠型的社会福利制度，促进社会慈善事业加快发展。围绕建设教育强市目标，将幼儿教育纳入国民教育体系，继续推进义务教育均衡发展，全面优化高中阶段教育，大力发展职业教育和终身教育，不断提升高等教育办学层次，2010年基本实现教育现代化，2012年力争筹建泰州大学。积极推动文化大发展大繁荣，启动实施“文化泰州建设行动计划”；着力构建覆盖城乡的公共文化服务体系，建成一批惠及城乡居民的文化馆（站、室）等基础设施工程；深化文化体制改革，推进文化艺术创新，开发一批优秀文化产品、打造一批省内领先和国内外知名的文化品牌、造就一批德艺双馨的文化人才，全市文化软实力明显增强。继续推进医疗卫生体制改革和资源整合，办好公益性医疗服务机构和骨干服务机构，显著改善农村和社区医疗卫生设施条件；建立覆盖所有城乡居民的基本医疗保障制度，逐步提高新型农村合作医疗参合标准和政府补贴标准；切实改善医疗服务质量，强化医疗机构管理，为群众提供安全、有效、方便、价廉的医疗卫生服务。稳定低生育水平，重视和解决人口老龄化问题，成为全省人口协调发展先进地区。加强社会管理，公共服务水平和公共产品供给能力进一步提高，社会治安综合治理和各类矛盾纠纷调处工作进一步强化，社会应急体系进一步完善，全市社会安定和谐、人民群众安居乐业。

5、加快建设生态文明，切实增强资源环境承载能力。坚持节约优先、环保优先，走生态文明发展道路，着力打造资源节约型、环境友好型社会。生态文明发展理念得到深入贯彻，全社会基本形成节约能源资源和保护生态环境的产业结构、增长方式、消费模式。以节水、节能、节地为重点的节约型经济体系建立健全，创建成国家节水型城市，万元GDP能耗年均下降4％以上，沿江开发区域每500米深水岸线投资强度在1亿美元或10亿元人民币以上。城镇生活污水处置设施、城市生活垃圾无害化处理设施进一步完善，水污染防治、大气污染治理和主要污染物排放控制成效明显，化学需氧量、二氧化硫排放量年均分别削减1.85％和0.51％，基本建成国家环保模范城市群。以生态保护和修复为主要内容的绿色泰州、生态泰州建设取得积极进展，2012年全市森林覆盖率达20％以上，城市绿化覆盖率45％以上，努力创建生态良好区域，确保创建成国家园林城市。

各位代表，全面达小康、建设新泰州，是历史赋予我们的重要使命。完成这一使命，需要统一意志，坚定信心；更需要开拓创新，埋头苦干。勤劳智慧的泰州人民在改革开放的道路上已经创造了不凡的业绩，在全面建设更高水平小康社会的新征程中，应当也完全能够谱写出更加绚丽的新篇章。泰州的明天一定会更加美好！

三、2008年工作建议

今年是全面贯彻落实党的十七大精神的起始之年，是实施“十一五”规划的关键之年，也是新一届政府的开局之年。做好今年工作，具有特别重要的意义。

今年政府工作的总体要求是：全面贯彻党的十七大和省委十一届三次全会精神，认真落实市委三届四次全会的决策部署，以科学发展观统领全局，转变发展方式，提升开放水平，统筹城乡建设，着力改善民生，促进社会和谐，努力实现经济社会又好又快发展，加快“全面达小康、建设新泰州”进程。

今年全市国民经济和社会发展主要调控目标建议安排为：地区生产总值可比增长12%以上，其中一产、二产、服务业增加值分别增长4%、14%和13%；财政收入增长16%，其中地方一般预算收入增长16％；全社会固定资产投资增长20%；社会消费品零售总额增长14%；自营出口增长12%；实际利用外资9亿美元；城镇居民人均可支配收入增长11%，农民人均纯收入增长9%；居民消费价格总水平涨幅不高于全省平均水平；城镇登记失业率控制在3.5%以内；万元GDP能耗下降4.4%。

今年，建议着重抓好八个方面的工作：

1、以增加农民收入为核心，扎实抓好“三农”工作。深入推进“5218工程”，加快优质水稻、弱筋小麦、“大佛指”银杏等优势农产品基地、产区建设，大力扶持畜禽养殖业发展，推进农业适度规模经营，新增高效农业面积20万亩；推动60家农业产业化龙头企业提速增效，新发展农村三大合作经济组织150家；健全农业科技服务体系、农产品质量标准体系和动植物疫病防控体系，新增无公害农产品、绿色食品、有机食品70个以上；实施化肥农药减量、畜禽养殖达标排放、水产生态养殖、秸杆综合利用和绿色泰州现代林业工程，积极发展生态农业。大力推进新农村建设，基本完成村庄规划编制，有序引导农民集中居住；继续实施“十、百、千”工程，新建市级全面小康先行村100个；扎实抓好农村垃圾处理、河道整治和道路、桥梁建设，切实加强农田水利基本设施建设和耕地保护；健全农村新“5+1”实事有效投入机制和工作推进机制，不断改善农村发展环境和农民生产生活条件；制订并实施新一轮“农村扶贫帮困三年计划”，将工作重点逐步向经济薄弱村和贫困农户延伸。继续加大新型农民培育力度，加快提升农民综合素质、向非农产业转移层次和就业创业能力，全年农业实用技术和创业培训15万人次以上。

2、以加快新型工业化为方向，继续推动工业结构优化升级。着力调高、调优、调轻产业结构，推动医药、机电、船舶、化工四大优势产业向高端发展，提高不锈钢制品等十大特色产业集群的产业关联度，继续改造和提升传统产业；鼓励和支持全市“50强”企业创新发展理念、建设主题园区、培育著名品牌，不断增强市场竞争力；进一步优化技改投资结构、促进银企合作、落实扶持政策，加快推进重大项目实施进度，年内组织实施亿元以上重大技改项目80个。着力推进自主创新，启动实施新一轮高新技术产业“双倍增”计划，加快建设新医药等十大自主创新特色基地，积极创建全国知识产权示范城市；继续实施“百企三创工程”，支持“一站两中心”建设，新认定市级以上高新技术企业50家；切实提高增量投入的科技含量，加快高新技术产品开发和产业化进程，滚动开发年销售3000万元以上新产品300个。着力促进节能减排，全面推进结构、技术和管理节能，突出抓好耗能大户节能、重点节能技改项目实施，加大高耗能设备淘汰力度，严格控制增量；筹建污染源自动监控中心和污染事故应急处置中心，扎实抓好重点污染源监管和企业违法排污、集中式饮用水源地安全等专项整治行动，组织开展循环经济试点，年内再关闭小化工企业40家。探索建立产业用地二次开发机制，开展土地利用总体规划修编工作，促进土地节约高效使用。

3、以实施“833工程”为抓手，努力促进服务业加速发展。优先发展生产性服务业，壮大泰州港综合物流园区、泰州城北物流园区、江苏三江现代物流中心等重点物流基地，大力发展第三方物流企业；抢抓国际服务外包转移机遇，招引高层次的研发机构、信息咨询机构和中介服务机构；积极发展外资银行、股份制商业银行和农村合作银行，确保江苏银行泰州分行、江苏长江商业银行如期挂牌，筹建浦发银行泰州分行，推动股份制商业银行向辖市延伸。加快发展新兴服务业，引进旅游业战略性投资者，重点抓好溱湖风景区、凤城河风景区、泰兴古银杏森林公园、靖江江心洲生态旅游区、兴化水上森林公园、引江河风景区等项目的建设和完善，建成国家等级旅游景区（点）和全国农业旅游示范点4个，旅游总收入力争超百亿元；发展连锁经营、配送中心等新型商贸业态，并积极向农村延伸和拓展；抓好人才柔性引进，发展具有泰州特色的软件产业、创意产业和娱乐产业。提升发展传统服务业，积极打造坡子街区域中心商业圈，有序推进府南商业区建设，加快培育品牌商贸企业和特色街区；扶持发展一批区域性“有根”市场，引导戴南不锈钢交易城、华东五金城等专业市场做大做强，规划建设再生资源交易市场；继续加强农村流通体系建设，积极发展为农服务社，让农民便捷消费、放心消费。

4、以强化重大项目招引为关键，进一步提升对外开放水平。紧紧围绕招引过亿美元重大项目，瞄准世界500强企业和国际行业龙头企业，继续强化产业链招商，重点引导外资更多地投向高端制造业和研发环节，全方位推进服务业开放，认真抓好现代农业推介，大力促进重点企业境内外上市，加快外资企业“本土化”进程。把加快开发区建设与增强自主创新能力、提高集约发展水平、培育园区产业特色结合起来，推进发展模式和园区功能创新，提升管理水平和产出效能。泰州医药高新技术产业园区着力在研发、制造、服务外包等方面取得突破，力争跻身国家级开发区行列。健全沿江用地、岸线等资源高效利用机制，落实最严格的环保制度，走好保护性开发的路子。推动自主品牌、自主知识产权的商品出口，探索建立农副加工产品出口促进机制和风险预警机制，扩大资源性产品和技术设备类产品进口。培育和发展外经重点企业，积极推动境外工程承包和劳务输出，鼓励有条件的企业开展境外投资；强化口岸基础设施建设，进一步提高口岸通关效率和通过能力，创建文明口岸。继续做好外事、侨务和港澳台事务等工作。深入推进跨江联动开发，加快锡常泰城市群融合步伐，拓展接轨上海的领域和空间。认真落实各项促进民营经济发展政策，进一步改善融资环境、优化中介服务，年内私营个体经济注册资本净增120亿元。

5、以完善功能、提升形象为着力点，突出抓好中心城市建设。进一步完善城市功能，实施市文化中心二期、凤城河风景区三期、高教园区三期、东进广场（烈士陵园改扩建）和市图书馆新馆等重点工程；推进老城区污水管网全覆盖，加快第四污水处理厂、垃圾中转站等环保设施建设；完善西北片城市防洪工程，实施东北片城市防洪工程和周山河整治工程。进一步拓展城市空间，启动周山河街区建设，基本建成园博园水系、路桥及主要服务设施；完成海陵路南延、鼓楼路南延和北延工程，启动高港区三大片区建设。进一步提升城市品位，有序推进“城中村”改造，加大城北街区改造和建工、春兰等老小区整治力度，加快城区工业企业“退城进区”步伐；启动五巷古民居保护和西城河两侧建设，完成高港生态公园二期工程。进一步强化城市规划和管理，开展新一轮城市总体规划修编工作，建立数字化城市管理模式，扎实推进新“五城同创”，创建成江苏省节水型城市。进一步加快城乡基础设施建设，协力推进泰州长江大桥、宁通高速公路扩容和江海高速公路建设，扎实抓好兴泰公路改扩建工程；大力推进区域供水工程，如期向姜堰、泰兴两市供应长江水；实施西气东输泰州天然气利用工程，继续完善电力、通信等基础设施。

6、以改善民生为重点，大力推进社会建设。继续抓好改善民生十大工程的实施和年度十件实事的办理，切实解决好影响民生的突出问题。把促进就业、再就业作为民生之本来抓，注重发展劳动密集型产业，积极实施“创业扶持工程”和“动态消除零就业家庭计划”，全年净增城镇就业人员3.5万人；认真贯彻落实《劳动合同法》，组织开展“劳动合同三年行动计划”，在市（区）全面建立劳动合同“网格化”、“信息化”管理模式，切实保障企业职工合法权益。把加强保障救助作为民生之基来抓，强化社会保险扩面征缴，扎实抓好全国城镇居民基本医疗保险试点，积极做好困难群体参保工作；大力推进农村社会基本养老保险，落实农民工灵活参保政策，将新型农村合作医疗参合最低标准提高到每人100元；积极发展社会救助、慈善事业，探索低保边缘人群、因病致贫和受灾家庭救助办法，加快老年服务设施和示范性养老机构建设，实施孤残儿童“蓝天计划”，建成市儿童福利院；市区新开工经济适用房22万平方米、租售并举经济适用房350套，着力解决城区低收入家庭住房困难。把发展各项社会事业作为民生之要来抓，切实加大义务教育投入和对家庭经济困难学生资助力度，在全面免除义务教育学杂费的基础上，实行城乡义务教育全部免费提供课本，进一步扩大资助家庭经济困难学生覆盖面，完善农村义务教育投入保障机制，有效化解农村义务教育债务，扎实抓好区域基本教育现代化迎评工作，继续推动各类教育协调发展；大力发展文化事业，办好首届泰州梅兰芳艺术节等一批重大文化节庆活动，筹办泰州市书画家优秀作品晋京展，备战第六届江苏省淮剧节，市梅纪馆和学政试院完成布馆并开放，加快市及市（区）文化馆、图书馆、乡镇文化站、村文化室和农家书屋建设；进一步完善城乡卫生服务网络，认真落实各项惠民医疗政策，强化药品价格监管和医德医风建设，逐步解决城乡居民看病贵、看病难的问题；落实公交优先发展政策，加大财政投入力度，加快公交车更新改造和场站建设步伐，迅速提高公交管理水平，确保年内市区公交状况有突破性改善；精心组织奥运火炬在泰州境内传递活动，推进有线数字电视整体平移和有线电视通组入户，加快人民防空与防灾一体化进程，组织开展全市第二次全国经济普查；积极发展史志档案、社会科学、气象、防震等事业，切实做好民族宗教、妇女儿童和残疾人等工作。

7、以打造文明、法治泰州为目标，切实加强精神文明和民主法制建设。全面推进文明泰州建设，扎实抓好文明城市创建工作，力争创成全国创建文明城市工作先进市；贯彻落实《公民道德建设实施纲要》，组织实施市民文明素质提升计划，不断放大“百姓”系列、“爱心”系列等品牌效应；大力开展城乡文明和谐共创活动，继续抓好文明社区、村镇、行业、单位、家庭等群众性精神文明创建。积极发展基层民主，全面编制村务公开目录，加强城市社区建设。扎实推进法治泰州建设，广泛开展建设“法治江苏合格县（市、区）”、“法治泰州建设合格乡镇（街道）”活动，80％以上的村（社区）达到民主法治村（社区）标准；认真抓好“五五”普法，发展法律援助事业，加强社会诚信建设。深入推进平安泰州建设，加强社会治安综合治理，建成全国第二批科技强警示范城市；健全信访工作责任制，排查、调处各类矛盾纠纷，预防和妥善处理群体性事件；加强市级应急指挥平台建设和基层应急管理，建成覆盖街道、乡镇及各类企事业单位的应急组织体系和预案体系；强化安全生产管理和监督，预防和遏制重、特大安全事故发生。

8、以构建服务型政府为主线，继续抓好政府自身建设。树立现代行政理念，综合运用规划、产业政策、技术和环境标准等手段，改进经济调节和市场监管方式，切实履行社会管理和公共服务职能。深化财税管理体制改革，建立健全公共财政体系，提升公共领域财政保障能力。继续抓好事业单位改革，深入推进“政社分开”、“管办分离”，促进社会中介组织规范有序发展。以学习贯彻党的十七大精神为重点，强化公务员教育和培训，创新交流和竞岗机制，不断提高公务员队伍整体素质。健全完善政府工作目标管理制度，加大行政问责和责任追究力度，强化行政监督，确保政令畅通，提高行政效能。继续深化行政审批制度改革，全面推进行政执法责任制，进一步优化经济发展软环境，建成泰州市民服务中心。稳步推进行政权力动态公开，完善新闻发言人制度和涉及群众切身利益的政府重大决策公众听证制度，保障群众的知情权、参与权、表达权和监督权。大力发展电子政务，突出抓好政府网站、行政电子监察系统和社区综合服务平台建设，进一步扩大网上服务领域。自觉接受市人大及其常委会的工作监督和法律监督，支持人民政协履行职能，主动听取民主党派、工商联、无党派人士和各人民团体的意见。全面落实廉政建设责任制，建立健全惩治和预防腐败体系，加强对政府机关和领导干部的监督，加大执法监察、廉政监察和财政资金使用监管的力度，强化对重点领域、重点部门和重点项目的审计。深入开展纠风和专项治理工作，依法查处各类违法违纪案件，树立清正廉洁的良好形象。

各位代表，新的形势催人奋进，新的征程任重道远。让我们高举中国特色社会主义伟大旗帜，深入贯彻落实科学发展观，在中共泰州市委领导下，开拓创新，锐意进取，真抓实干，奋发有为，沿着党的十七大指引的道路奋勇前进，为全面建设更高水平的小康社会作出新的更大的贡献！
